# The Use of Different Commercial Mineral Water Brands to Produce Oil-In-Water Nanoemulsions

**DOI:** 10.3390/molecules25030603

**Published:** 2020-01-30

**Authors:** Pedro A Rocha-Filho, Antonio D. Monteiro, Luciana C. Agostinho, Marina P. A. Oliveira

**Affiliations:** School of Pharmaceutical Sciences of Ribeirão Preto, Department of Pharmaceutical Sciences, University of São Paulo, Ribeirão Preto 14040-903, SP, Brazil; antonio.domenico.monteiro@usp.br (A.D.M.); luagostinho@usp.br (L.C.A.); mpaoliveira@usp.br (M.P.A.O.)

**Keywords:** nanoemulsion, stability, water, electrolytes

## Abstract

Nanoemulsions are submicron-size colloidal systems that have the ability to encapsulate, protect, and deliver active ingredients. They have been used in the pharmaceutical, cosmetics, and food industries to improve the absorption of drugs by the skin or via the gastrointestinal tract, aide in food conservation, and treat skin problems. To proper formulate a nanoemulsion, it is important to know the characteristics of its components (aqueous and oil phases, surfactants and additives), as well as the influence on the production method that will be used. This study investigates the influence of aqueous phase composition, stability and particle size in an oil-and-water nanoemulsion formation. By using a low energy method, the purified water was exchanged for different commercial mineral water and saline solutions, and the results of stability, particle size, pH and conductivity tests, were compared. These results show that the minerals present in commercial waters may alter the particle size, pH and conductivity values of nanoemulsions, as well as their stability.

## 1. Introduction

Nanoemulsions (NEs) are colloidal systems in which particle sizes are in the nanometric scale and which draw attention due to their capacity to encapsulate, protect and deliver active ingredients of the formulation. The small size of the particle means the surface area-to-volume ratio is large, which may be advantageous for applications in food, cosmetics and pharmaceutical products due to their ability to incorporate hydrophilic and lipophilic drugs and a low occurrence of irritation, a high interfacial area-to-volume ratio, and an increased drug penetration through the skin, as compared to microemulsions [1,2]

Though this system is thermodynamically unstable, the selection of ingredients and preparation method are essential factors to produce systems that will remain stable for a long period of time [3]. The particle size and polydispersity index (PdI) of an NE are characteristics that affects its stability, rheology, appearance, color, and texture [4].

NEs may be oil-in-water (O/W), if the internal phase is lipophilic, or water-in-oil (W/O), if the internal phase is hydrophilic. NEs may be produced by using high or low energy methods. High energy methods generally involve mechanical devices that are capable of producing intense disruptive forces to break down the droplets of an emulsion into nanometric size ones, like high pressure homogenizers, ultrasonic devices or microfluidizers. Low energy methods rely on spontaneous formation or a change in the conditions of the formulation during preparation, such as temperature, the oil-to-surfactant ratio, and composition. These methods include spontaneous emulsification, phase inversion temperature, phase inversion composition, and the emulsion inversion point, although some authors consider the last two methods to be the same [1,5]. 

The emulsion inversion point method has been used to produce an NE with rice bran oil, non-ionic surfactants, and commercial mineral waters due to the fact that this method requires a lesser input of energy and enables the production of an NE with small droplets in a single batch [5]. The principle behind the method is the transformation of an oil-in-water (O/W) emulsion into a water-in-oil (W/O) NE, as water is titrated into the system that contains oil and surfactants through a catastrophic phase inversion that reduces the size of the droplets [5].

To formulate an NE, it is important to consider the preparation method that will be used and to study the characteristics of the oil (or oils), the nature and proportion of surfactants, and the composition of the aqueous phase. The aqueous phase is typically constituted by purified water, but it may also contain other polar components that may interfere with characteristics, such as polarity, interfacial tension, pH, and ionic strength, that impact the formation and stability of the NE. Those changes may also interfere with the ability of the surfactants to form droplets [1,6]

Gaonkar (1992) studied the effects of salt addition in colloidal systems and found that salt addition resulted in a lower surface tension than an emulsion that was produced only with water. The addition of an increasing amount of salt in colloidal systems may result in instability or interferences in the electrostatic repulsion between the globules. However, the addition of small quantities of salt may increase electrostatic repulsion to surpass the hydrophobic attraction of Van der Waals forces until a critical value for that specific system, beyond that of the electrostatic repulsion, is no longer able to ensure the repulsion between globules, thus leading to coalescence [6,7].

In this work, we used the emulsion inversion point method to produce an NE with rice bran oil and non-ionic surfactants waters to study the influence of the aqueous phase in order to determine changes in particle size, PdI, and stability by using mineral waters of different compositions. 

## 2. Results and Discussion

### 2.1. Nanoemulsion Preparation

NEs were prepared as those in a study by Bernardi (2011): by using the surfactant mixture sorbitan monooleate/PEG-30 castor oil at 10% (*w*/*w*) [4]. Rice bran oil (10% *w*/*w*) was used as its oil phase and aqueous phase was composed only by water (80% *w*/*w*). Six different NEs were prepared, five of them by using a different brand of mineral water and one by using purified water that was freshly obtained by reverse osmosis. The composition of minerals of the commercial waters, as declared by their labels, is presented on Table 1.

The final NEs were macroscopically evaluated 24 h after preparation, and no signs of instability were perceived. Because all NEs were considered macroscopically stable, samples were submitted to centrifugation, and none showed signs of instability. 

### 2.2. Heating–Cooling Cycle (HCC)

The NE samples were evaluated to determine their pH, conductivity, particle size and PdI before and after the heating–cooling cycle (HCC). All of the samples remained macroscopically stable during and after the cycle. 

#### 2.2.1. pH Value

The pH values of NEs that were prepared with commercial mineral waters were slightly acidic before the cycle, with the exception of mineral water (W4), which had a higher pH value (7.14). This characteristic may have been related to the higher content of HCO_3_^−^, declared by the label as 163.91 mg/L. The NEs with the lower pH value were prepared with W3, which had a pH = 6.17. After the cycle, the NEs prepared with W2 showed a slightly increase in pH value (6.45–6.52) of around 1%. With the exception of the NE that was prepared with W2, those prepared with W4 and W1 showed the lowest variation of pH value of around 10%; this may have been caused by the higher concentration of HCO_3_^−^, which may have created a buffer effect. The NE that was prepared with W5 showed a higher decrease (6.96–5.49), but its pH value remained compatible with skin. The W5 composition showed a higher content of Na^+^ and SO_4_^2−^. The pH values of the NEs before and after the HCC are presented in Table 2.

#### 2.2.2. Conductivity

The conductivity value of an NE is related to the characteristics of its continuous phase, and its alteration may indicate instability occurrence. An increase in a conductivity value may be related to coalescence, while a decrease may indicate aggregation [8]. Conductivity values and their variations before and after the HCC are shown in Table 3. 

All the NEs except for those prepared with W4 and W5 showed an increase in their conductivity value, with the W3 NE being the smaller variation (190.3–190.45). The W4 preparation showed the higher variation (289.9–155.94), despite presenting no signs of macroscopic instability. 

#### 2.2.3. Particle Size and PdI

Particle size measurements are important to determine if a colloidal dispersion is an NE (particle size in nanometric scale) and may be used to show system stability through time. The PdI is a dimensionless number that is correlated to the mono or polydispersity of the particle size populations of the NE. In this work, the distribution report used was determinate by intensity, and size is presented as z-average. Particle sizes and PdI values are presented in Table 4. 

Table 4 shows that, before the cycle, almost all NEs presented particle sizes from 53 to 63 nm, a PdI below 0.3, and monomodal dispersion. The exception was that prepared with W2, which had a second population around 4854 nm (1.3%), and purified water, which had a second population around 16.28 nm (2.8%). After the cycle, just the W3 preparation maintained monodispersity; all the other formulations showed two populations of droplet sizes. 

Considering that Dynamic Light Scattering measures the hydrodynamic diameter of a particle, i.e., the diameter of the NE globule itself plus solvation layer and any ions or molecule that may be interacting with the globule, differences in electrolyte concentrations in the aqueous phase and the interactions between particles may interfere with the measured size. The stabilization of NEs occurs mainly via steric mechanisms, but electrostatic forces may be considered. As the aqueous phase of NEs that are prepared with different waters have different concentrations and types of electrolytes, electrostatic forces may influence the size of the particle, as measured by DLS, and the stability of the NE. The W4 and W5 preparations, which showed the higher increases in particle size after the HCC, were the waters with a higher concentration of HCO_3_^−^: 163.91 and 83.40 mg/L, respectively. This may have corresponded to a correlation between the presence and influence of this electrolyte on the behavior of NEs in the stability tests. 

#### 2.2.4. Thermal Stress

The NE prepared with W4 showed a phase separation after thermal stress. All the other NEs remained stable after the test. 

#### 2.2.5. NEs with Modified Aqueous Phase

The W4 preparation showed the highest alteration in particle size and conductivity, as well as a small decrease in pH value and a phase separation after thermal stress. The mineral water composition (Table 1) showed that its main electrolyte was HCO_3_^−^. The second highest difference in particle size was that of W5, which also had a high concentration of HCO_3_^−^. Fornaguera et al. (2016) prepared NEs by using different electrolyte concentrations to study the influence of electrolytes in the formation and stability of NEs, and they found decreases of droplet size [9]. By using the same concentrations as a template, new NEs were prepared that had the same formulation (see Section 2.1.) but these included NaHCO_3_ (NE*a) or NaCl (NE*b) in the aqueous phase at 0.03, 0.08 and 0.16 mol/L.

NE*s were submitted to the HCC and evaluated to determine their particle size and PdI before and after the HCC. All the measurements were made in triplicate, and the results are presented Table 4 in terms of z-average (nm).

NE*a and NE*b showed a slight increase in particle size, when compared to the NE that was produced only with purified water, before the HCC. NE*a showed less variation in particle size among the tested concentrations than NE*b, which displayed a particle size that increased with higher concentration of NaCl. The particle sizes obtained before the HCC for NE*a and NE*b were compatible to the size obtained when using mineral waters (Table 5). After the HCC, however, the NEs that were produced with mineral waters showed a much higher increase in particle size, which suggested that the presence and combination of electrolytes in the aqueous phase may influence particle size and stability of an NE. 

The PdI value of NE*a decreased with the higher concentration of NaHCO_3_, keeping values under 0.1, thus indicating a monodispersed NE. These results suggest that the addition of NaHCO_3_ in the aqueous phase may have improved stability, considering that the size and PdI values of NE*a showed only a small variation before and after the cycle. The result differs from observed for the NE that was produced with W4 that presented a higher increase in particle size.

NE*b showed a non-linear variation of the PdI among different concentration of NaCl. When comparing values before and after the HCC, higher concentration of NaCl led to a decrease in the PdI value but increase in particle size. 

## 3. Materials and Methods 

Rice bran oil, surfactants sorbitan monooleate, PEG-30 castor oil, purified water and commercial mineral waters of different brands named W1, W2, W3, W4 and W5 were used for this study.

### 3.1. Methods

#### 3.1.1. Nanonanoemulsion Preparation

NEs were prepared by using the Emulsion Phase Inversion (EPI) method in which the aqueous and oil phase were heated separately until reaching 75 ± 2 °C. The aqueous phase was then poured over the oil phase under agitation (650 rpm) until it reached room temperature (25 ± 2 °C) [10]. 

#### 3.1.2. Preliminary Stability Evaluation

The NEs were macroscopically evaluated, after 24 h of preparation, for signs of instability (phase separation, flocculation or creaming). Five milliliters of the NEs was centrifuged for 30 min at 300 rpm, and then they were evaluated again for signs of instability [11,12].

#### 3.1.3. Heating–Cooling Cycle (HCC)

The NE samples (50 mL) were submitted to cycles of 24 h at 4 ± 2 °C and then 24 h at 45 ± 2 °C. The centrifugation, pH and conductivity tests were conducted after 24 h of preparation and after 3° and 6° cycles (6° and 12° days, respectively) [8].

#### 3.1.4. Particle Size and PdI

The particle size and PdI were evaluated by using dynamic light scattering (Zetasizer Nano ZS) with samples diluted at 1:100 and homogenized.

#### 3.1.5. Thermal Stress

The NE samples were heated in a thermostatic bath, from 40 to 85 ± 2 °C, increasing temperature by 5 °C each 30 min. Then the samples were analyzed for signs of macroscopic instability.

## 4. Conclusions

Mineral waters of different composition were used as aqueous phases to produce NEs while using the same basic formulation. The presence of electrolytes may influence the interaction of NE droplets, improving their stability or causing instability issues. To assess the characteristics of the produced NEs, pH, conductivity values, particle size, and PdI values were analyzed. From the results of the HCC test, the NE that was prepared with W1 showed a higher stability, a low variation of pH and conductivity values, and a smaller increase in particle size compared to purified water. The W4 preparation had the higher concentration of HCO_3_¯, which contributed to a higher initial pH value of the NE, but this preparation also showed the highest variation in conductivity value and particle size. The correlation of the concentration of the monovalent ions Na^+^, Cl^−^ and HCO_3_^−^ that are present in commercial waters and its effects on NE characteristics were investigated. The addition of these electrolytes (as NaCl and NaHCO_3_) in the aqueous phase of the NEs increased their particle size when compared to the NE that was prepared just with purified water. The NEs prepared with NaCl showed a correlation between the higher concentration of the salt and an increase in particle size, but they maintained their nanometric size. The NEs that were prepared with NaHCO_3_ showed only a small variation in particle size and a smaller PdI, indicating a more stable and monodisperse system. The presence of electrolytes may influence the interaction of droplets, improving stability or causing instability issues. Particle size, as measured by DLS, may be influenced by the composition of the aqueous phase. When preparing an NE, aqueous phase composition must be carefully studied, because its elements may interfere in the stability of the final product. 

## Figures and Tables

**Table 1:** Chemical composition of commercial waters, as declared by their label.

Chemical Composition (mg/L)					
	W1	W2	W3	W4	W5
HCO_3_^−^	68.0	23.45	29.95	163.91	83.40
Ca^2+^	11.70	5.95	5.73	32.10	14.291
Na^+^	2.90	0.899	4.64	3.32	7.892
Mg^2+^	5.39	2.130	0.896	15.60	4.556
Cl^−^	0.61	1.52	0.86	11.78	0.03
K^+^	3.52	3.69	2.39	3.38	1.058
SO_4_^2−^	3.88	-	0.53	1.06	4.19
F^−^	0.27	0.06	0.09	0.10	0.15
Sr^2+^	0.141	0.037	0.121	0.022	0.07
PO_4_^3−^	-	-	0.25	-	0.30
Ba^2+^	0.06	0.096	-	0.063	0.025
NO_3_^−^	1.30	11.00	5.58	4.44	-

**Table 2:** pH values before and after the heating–cooling cycle (HCC).

	pH	
	Before	After
Purified water	6.28 ± 0.15	5.17 ± 0.11
W1	6.5 ± 0.12	5.82 ± 0.18
W2	6.45 ± 0.05	6.52 ± 0.14
W3	6.17 ± 0.04	5.4 ± 0.14
W4	7.14 ± 0.07	6.41 ± 0.08
W5	6.96 ± 0.06	5.49 ± 0.03

**Table 3:** Conductivity values before and after the HCC.

	Conductivity (μS/cm)	
	Before	After
Purified water	174.7 ± 0.58	194.3 ± 0.28
W1	226.8 ± 1.69	246.1 ± 1.39
W2	219.7 ± 1.28	340.6 ± 1.06
W3	190.3 ± 2.17	190.45 ± 0.54
W4	289.9 ± 1.80	155.94 ± 0.76
W5	245.2 ± 2.22	222.8 ± 0.94

**Table 4:** Particle size (nm) and polydispersity index (PdI) ** before and after the HCC *.

	Before HCC *		After HCC *	
	z-Average (nm)	PdI **	z-Average (nm)	PdI **
Purified water	63.56 ± 0.38	0.174 ± 0.003	85.02 ± 0.85	0.375 ± 0.009
W1	54.17 ± 0.27	0.167 ± 0.007	94.38 ± 2.69	0.581 ± 0.086
W2	59.53 ± 0.32	0.197 ± 0.038	326.16 ± 8.48	0.412 ± 0.064
W3	53.28 ± 0.29	0.181 ± 0.004	197.16 ± 2.41	0.236 ± 0.007
W4	56.62 ± 0.61	0.163 ± 0.009	370.53 ± 8.73	0.342 ± 0.033
W5	63.21 ± 0.58	0.268 ± 0.032	409.26 ± 7.78	0.383 ± 0.026

* HCC = heating–cooling cycle; ** PdI = polydispersity Index.

**Table 5:** Particle size (nm) and PdI of NE*s (nanoemulsions produced with NaHCO_3_ and NaCl) before and after the HCC.

	Before HCC		After HCC	
	Particle Size (nm)	PdI	Particle Size (nm)	PdI
Purified water	53.23 ± 1.08	0.142±0.007	51.62±0.83	0.189±0.006
NE*a [0.03]	63.51 ± 0.25	0.087 ± 0.006	63.51 ± 0.33	0.098 ± 0.009
NE*a [0.08]	64.14 ± 0.17	0.072 ± 0.005	65.12 ± 0.37	0.083 ± 0.011
NE*a [0.16]	62.83 ± 0.21	0.061 ± 0.011	63.76 ± 0.42	0.068 ± 0.015
NE*b [0.03]	63.42 ± 0.35	0.14 ± 0.004	67.12 ± 0.49	0.210 ± 0.008
NE*b [0.08]	74.01 ± 0.28	0.197 ± 0.03	79.02 ± 0.32	0.185 ± 0.015
NE*b [0.16]	77.89 ± 0.15	0.195 ± 0.09	88.95 ± 0.20	0.106 ± 0.008

NE*a: NE prepared with NaHCO_3_ in its aqueous phase at 0.03, 0.08 and 0.16 mol/L. NE*b: NE prepared with NaCl in its aqueous phase at 0.03, 0.08 and 0.16 mol/L. Particle size: z-average
